# Dental Press Journal of Orthodontics: One year later, and more growth

**DOI:** 10.1590/2177-6709.22.4.009-010.edt

**Published:** 2017

**Authors:** David Normando

**Affiliations:** 1 Adjunct professor, Universidade Federal do Pará (UFPA), Faculdade de Odontologia, Belém, Pará, Brazil. Coordinator, Universidade Federal do Pará (UFPA), programa de pós-graduação em Odontologia, and ABO-Pará, Curso de Especialização em Ortodontia, Belém, Pará, Brazil.

*“A dream you dream alone is only a dream. A dream you dream together is reality.”*
Yoko Ono, Japanese artist

In last June SCImago launched the 2017 cites per doc of the journals that make up its bibliographic database. As is the case with the impact factor, this bibliometric index is calculated each year and is based on the average number of citations of articles published in a given periodical. Although this bibliometric methodology has drawn criticism it is believed that the more often a recently published article is cited, the greater its impact within the scientific context. 

And despite the tremendous growth it has experienced in recent years orthodontics is still not among the specialties with the greatest impact in dentistry. However, in 2016, thanks to a rise in the number of citations from some periodicals, among which is the Dental Press Journal of Orthodontics (DPJO), we rank amongst the fastest growing specialties in the dental field.

**Figure 1:**
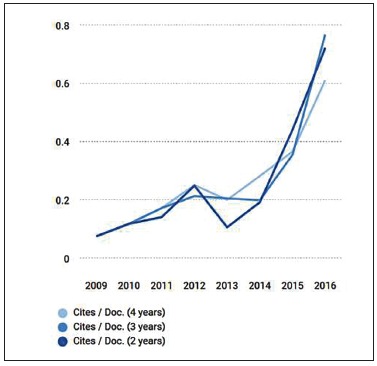
Citations per document in the SCImago database for the Dental Press Journal of Orthodontics.

DPJO, indexed to Scopus’s SCImago database, received its first cites per doc in 2009, when it was still only published in Portuguese. As of 2010, DPJO launched its English version.^1^ In June 2016, we received citation data from 2015, when we achieved an index of 0.44 - a 130% increase over the previous year. This significant growth is ratified by the data released in 2017, according to which we achieved a 2-year cites per doc of 0.72, denoting an increase of 64% over 2016 (Fig. 1), and nearly 7 times for the last triennium.

In addition to the invaluable cooperation of researchers, reviewers and the editorial board, there are three other fundamental factors that have enabled DPJO to sustain such dramatic, unremitting growth. The first is the adjustment in the publication flow by reducing the time spent between submitting and publishing an article, and especially publishing a smaller, more selective number of articles. While in 2013 we published 137 articles, there were 108 in 2014, 93 in 2015 and 78 in 2016, an average reduction of 17% per year in the last three years. By doing so, we managed to decrease the equation’s denominator through a more stringent selection process. 

The second reason was the indexing of DPJO to the PubMed database in 2013, making it more easily accessible to researchers worldwide. This insertion improved the chances of citations, thereby increasing the equation’s numerator, which was further reinforced by DPJO’s indexation to PubMed Central, yet a third important achievement. PubMed Central allows direct and free access to articles published from 2014, through the link www.ncbi.nlm.nih.gov/pmc/journals/2644/. Together, these achievements made possible a leap from 70 citations in 2013 - before PubMed indexation - to 136 in 2015^2^ and 232 in 2016, an average increase of 77% a year for the past 3 years. These figures, according to the SCImago database, indicate that currently DPJO is one of the 5 orthodontic journals with the highest number of citation/3 years worldwide. For 2018, our goal is to rank among the top 4, surpassed only by the classics *American Journal of Orthodontics and Dentofacial Orthopedics*, *The Angle Orthodontist* and *The European Journal of Orthodontics*.

These numbers may surprise many, but not those who work indefatigably for the growth of this major vehicle of orthodontic science communication. It is a dream come true for many, but also the offspring of detailed planning, as reported in a previously published editorial.^2^ As well as being among the 5 journals with the highest number of citations in world orthodontics, the SJR ranking of the SCImago database - which weighs the average number of citations received in 2016 by the number of articles published in the three previous years (2013, 2014 and 2015) - places the DPJO as the 2nd Brazilian dental journal and the 37th of all Brazilian science, among the 344 Brazilian journals indexed to the database. Three years ago, we were the 4th Brazilian dental journal and occupied the 173rd position among Brazilian journals.

 The next question is whether there is still room for growth. For 2018, we do not envisage a reduction in the number of published articles (the equation’s denominator). However, if we allow our expectations to be based on the average increase in the number of citations in the last two years, the 2-year cites per doc of the DPJO should be around 1.01. Too accurate? No, for scientific reason. Yes, for our dreams. For we want more.

We are fine-tuning for new achievements, but never losing sight of the mission that came to light at the very dawn of this journal: to promote orthodontic science for the clinical orthodontist. No doubt there were many hurdles along the way, but many of those hurdles were overcome by the three editors who preceded me. I am following in their footsteps. In between those hurdles there are encouraging numbers that nurture our dreams. But it is only when we are awake that they materialize. And the task is rendered much lighter when we can rely on so many helping hands and minds!

David Normando - Editor-In-Chief (davidnormando@hotmail.com)
